# A bibliometric analysis of anxiety and depression among primary school students

**DOI:** 10.3389/fpsyt.2024.1431215

**Published:** 2024-08-02

**Authors:** Jian Nan Fu, Wen Bing Yu, Shuo Qi Li, Wen Ze Sun

**Affiliations:** ^1^ Teaching Center of Fundamental Courses, Ocean University of China, Qingdao, Shandong, China; ^2^ Institute of Sports Science, Nantong University, Nantong, Jiangsu, China

**Keywords:** anxiety, depression, primary school students, bibliometrics, CiteSpace, VOSviewer

## Abstract

**Background:**

Rising anxiety and depression in primary school students adversely affect their development and academics, burdening families and schools. This trend necessitates urgent, focused research within this young demographic. This alarming trend calls for a systematic bibliometric analysis to develop effective preventative and remedial strategies

**Objectives:**

This study aims to identify and analyze the prevailing research hotspots and emerging trends concerning anxiety and depression in primary school students, thereby furnishing a foundational reference for future academic endeavors in this area.

**Methods:**

This study uses the Web of Science (WOS) Core Collection database as the data source, focusing on literature published between 2013 and 2023 concerning anxiety and depression in primary school students. An initial search identified 1852 articles, which were then manually screened to exclude duplicates, conferences, announcements, and unrelated literature, resulting in 1791 relevant articles. The analysis, executed on December 31, 2023, employed CiteSpace and Vosviewer tools to assess various bibliometric indicators including authorship, country, institutional affiliations, publication trends, keyword frequency, and citation analysis.

**Results:**

The analysis revealed a corpus of 1,791 English-language articles, with a discernible upward trend in publications over the decade. The USA and China were the leading countries in this field, with 482and 272 papers, respectively. The research predominantly addresses the etiological factors of anxiety and depression, various intervention strategies, and the comorbidities associated with these conditions in the target population. Key research focuses have been identified in areas such as suicidal thoughts, bullying in schools, the impact of COVID-19, mindfulness interventions, and anxiety related to mathematics. Future research is projected to increasingly focus on the effects of mathematics anxiety on the psychological and behavioral outcomes in students.

**Conclusion:**

This study provides a critical visual and analytical overview of the key research areas and trends in the field of anxiety and depression among primary school students. It underscores the necessity of concentrating on the underlying causes and potential interventions. Such focused research is imperative for mitigating the mental health challenges faced by young students and enhancing their educational and developmental outcomes.

## 1 Introduction

Depression and anxiety are prominent contributors to illness and disability in adolescents (1). In recent years, the prevalence of anxiety and depression among primary school students has been on the rise due to various factors such as family stress, social pressure and academic burden, which has become a global concern demanding significant attention. Research indicates a concerning upward trend in depression rates, escalating from 18.4% in 2000 to 26.3% in 2016 (2). A 2023 meta-analysis in China revealed that during the COVID-19 pandemic, both depressive and anxiety symptoms were prevalent at rates of 31% (3). Depression is expected to become the highest-burden disease worldwide by 2030 (4). Given that primary school students are undergoing crucial stages of emotional development, addressing psychological issues during this period is paramount, as they can have profound and enduring effects on their lives. The research indicates that the emergence of depression during primary school can lead to a series of irreversible adverse consequences, including social disorders, substance abuse (particularly alcohol abuse, internet addiction, and smoking), as well as severe obesity (5). However, it is essential to recognize that students’ anxiety and depression issues are not static; they are dynamic processes influenced by various factors over time. For example, during the COVID-19 pandemic, the probability of anxiety and depression in primary school students increased significantly, primarily due to social isolation (6). Recently, the anxiety and depression of primary school students in China have been attributed to excessive academic burden and insufficient sleep (7). Additionally, there is heterogeneity among different groups. Studies have shown that anxiety and depression levels are generally higher among rural primary school students compared to their urban counterparts (8). These differences are closely related to various factors, including family economic status, the availability of educational resources, family support systems, and differences in social environments (9, 10). Thus, investigating the problems and interventions related to anxiety and depression in primary school students is vital for promoting their mental health, supporting their healthy development, and contributing to the harmonious progress of society.

There is a need to investigate specific hypotheses regarding the underlying mechanisms of these psychological issues and the efficacy of targeted interventions. Possible hypotheses for this study include: (1) family stress, social pressure, and academic burden significantly contribute to the prevalence of anxiety and depression among primary school students; (2) early intervention and targeted therapeutic approaches can substantially reduce the prevalence and severity of anxiety and depression. Additionally, cultural factors must be considered in the study of anxiety and depression among primary school students. Cultural considerations encompass family expectations, societal norms, and the stigma surrounding mental health. In many Chinese families, a strong emphasis on academic success leads to significant pressure on primary school students (11). Societal norms often discourage open discussion of mental health issues, resulting in a lack of awareness and support, thereby exacerbating feelings of isolation and helplessness. The stigma surrounding mental health issues can prevent students from seeking help, creating a cultural barrier that must be addressed in any effective intervention strategy. By incorporating these cultural considerations, this study aims to provide a comprehensive understanding of the factors contributing to anxiety and depression among primary school students and to develop interventions that are both effective and culturally sensitive.

VOSviewer and CiteSpace are advanced bibliometric tools that help researchers visualize complex data from scientific publications. VOSviewer uses Visualization of Similarities mapping to identify and display relationships between different scientific entities like countries, organizations, and keywords (12). Similarly, CiteSpace applies network algorithms to analyze literature trends and co-citation patterns, offering visual maps that highlight key themes and development trajectories in a field (13). Currently, this field lacks metrological studies. This study utilizes CiteSpace (6.3.R1 advance) and the VOSviewer to conduct a visual analysis of primary school students’ depression and anxiety, aiming to provide a foundational reference for future theoretical and practical research.

## 2 Materials and methods

### 2.1 Data source and search strategy

**Figure 1:**
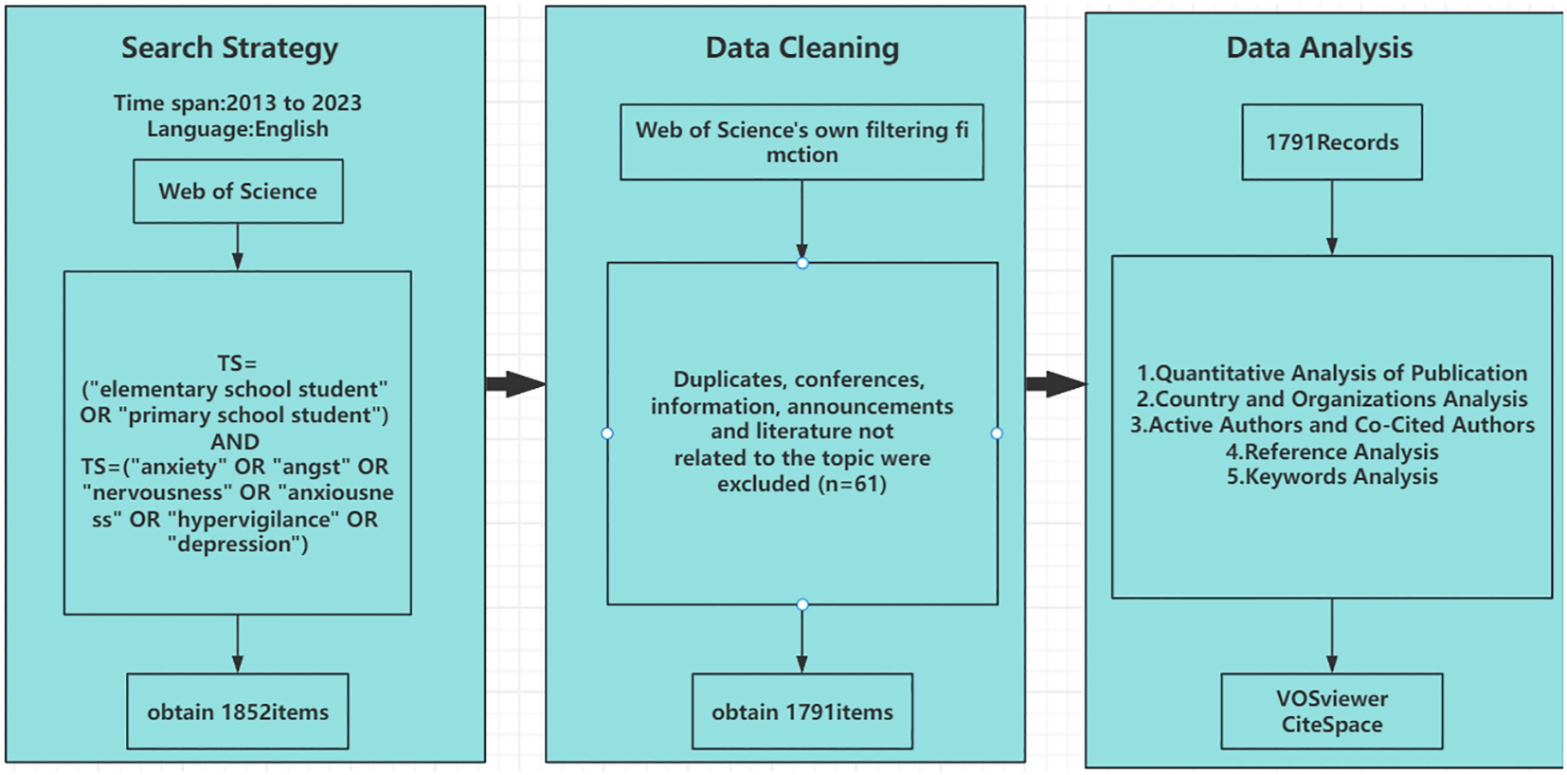
Data retrieval flow chart.

The Web of Science was utilized as the primary data source for this study, with literature being specifically collected from the core collection of the Web of Science database, spanning the period from January 1, 2013, to December 31, 2023. We used the PubMed database and queried subject terms through MeSH terminology, confirming the search terms based on expert knowledge. The search strategy employed was: TS=(“elementary school students” OR “primary school students”) AND TS=(“anxiety” OR “anxious” OR “nervousness” OR “apprehension” OR “hypervigilance” OR “depression”). The initial search yielded 1862 documents. To ensure the quality and reliability of our literature review, we used the filtering functions of the Web of Science to exclude certain document types, retaining only articles and reviews. We manually excluded 61 documents that were duplicates, off-topic, or did not meet the predefined selection criteria through a review of authors, keywords, titles, and abstracts. Ultimately, 1791 articles were included in this study. For further analysis, the selected literature was saved in “full record and cited references” format as txt files for subsequent bibliometric analysis using CiteSpace and VOSviewer. The article screening process is illustrated in **Figure 1**. The specific exclusion criteria were: (1) articles not related to anxiety and depression in elementary school students (ages 6-14); (2) document types other than articles and reviews.

### 2.2 Data extraction

A standardized search strategy was employed by two researchers to extract literature, with synonymous keywords being consolidated; for example, variations of “primary school students” were standardized to “primary school students”, and different forms of “depression” were unified to “depression”. Discrepancies in keywords were resolved through discussions among the researchers and, when necessary, with the consultation of a third party. Literature was screened in batches according to the inclusion criteria to identify eligible studies. Authors were included regardless of their rank, and their contributions were referenced for the number of publications in this study.

### 2.3 Visualization analysis method and bibliometric analysis

Software tools such as CiteSpace (version 6.3.R1 advance) and VOSviewer were utilized for the bibliometric analysis of literature concerning the mental health of primary school students. Knowledge graphs were generated by these tools, focusing on word frequency, clustering, and citation analysis across modules such as authors, countries, institutions, keywords, and references. Leading authors, countries, and institutions in the field over the past decade were identified by the analysis. Additionally, dominant themes and burgeoning frontiers in the research of primary school students’ mental health were explored, offering insights into prospective research trajectories.

## 3 Results

### 3.1 Overall characteristics of publications

**Figure 2:**
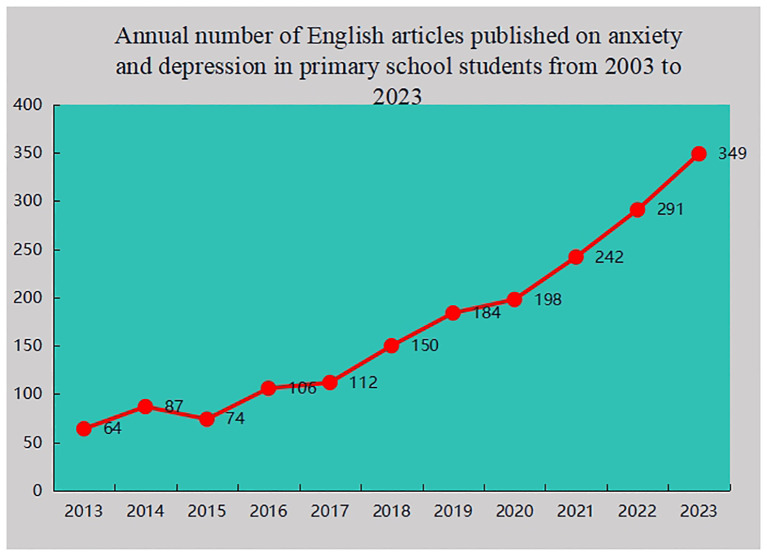
Annual number of English articles published on anxiety and depression in primary school students from 2003 to 2023.

As illustrated in **Figure 2**, the publication trend of articles has been segmented into two periods. In the initial phase (from 2013 to 2017), a slow fluctuation in the number of publications was observed, indicating modest scholarly interest in the mental health of primary school students. During this period, research was primarily focused on exploring basic concepts without extensive in-depth investigation. However, in the subsequent phase (from 2017 to 2023), a rapid increase in publications was noted, signifying that anxiety and depression among primary school students have emerged as significant research topics, with related studies entering a phase of rapid development.

Moreover, a consistent rise in the overall volume of literature on anxiety and depression among primary school students from 2013 to 2023 was observed, particularly notable between 2017 and 2023. This trend suggests a likely continuation in the increase of relevant literature in this area, reflecting the growing academic focus in recent years on the study of anxiety and depression among primary school students and underscoring its increasing relevance.

### 3.2 Analysis of authors and co-cited authors

**Figure 3:**
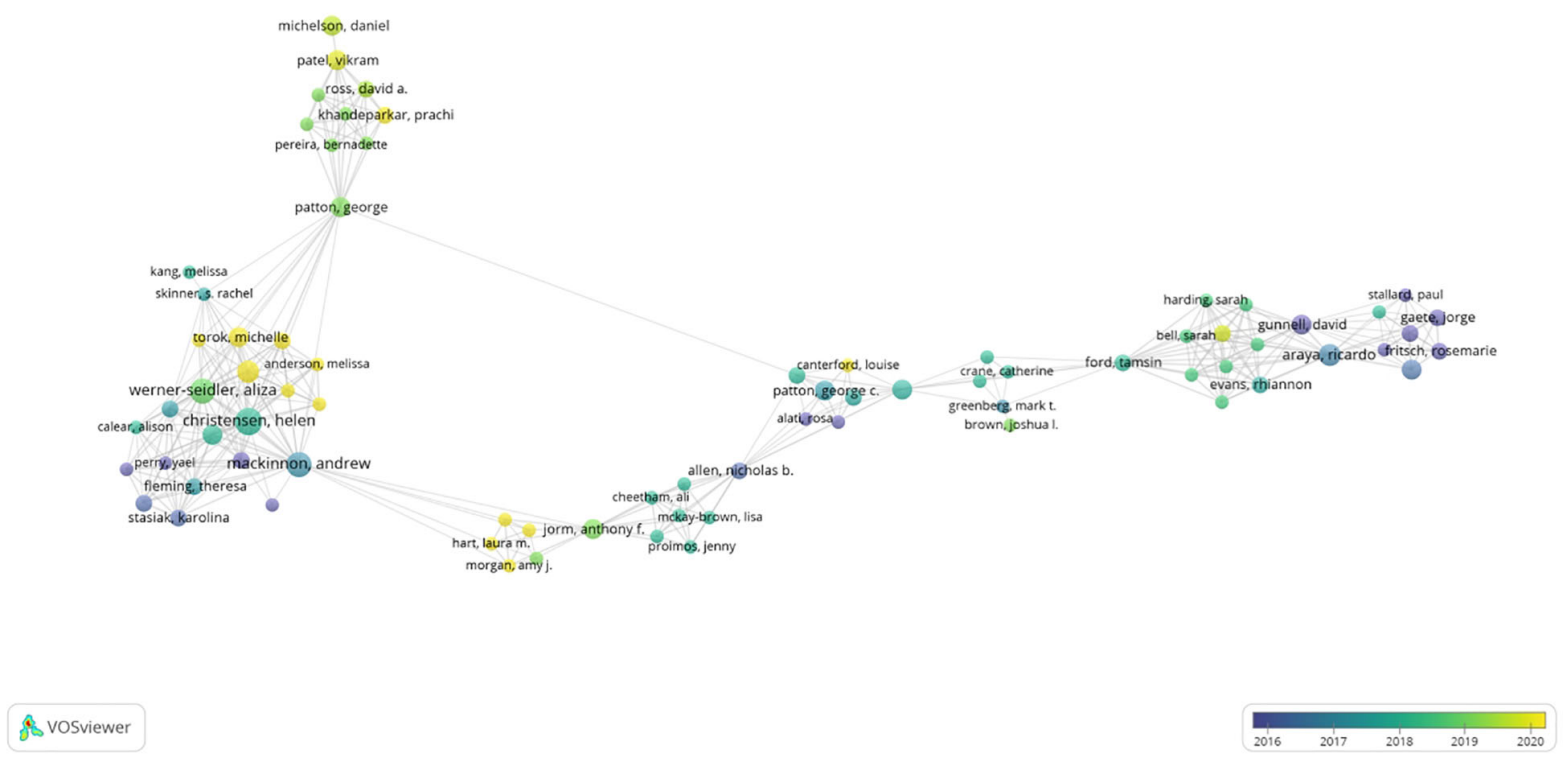
The visualization of authors on research of anxiety and depression among primary school students.

Putwain, David W. (n=7), and Ginsburg, Golda S. (n=7), are the two most prolific authors. There were two authors who were co-cited more than 150 times: COHEN J. (n = 167) and HU LT. (n = 151) (**Table 1**). These authors can be considered leaders in the field of anxiety and depression research among primary school students. In the visual knowledge map of co-authors, depicted in **Figure 3**, the size of author nodes is shown to be proportional to their publication output, and the connecting lines represent collaborations between authors. Significant collaborative networks among several researchers have been identified. For instance, collaborations between Tim Dalgleish, Mark T. Greenberg, and Darren Dunning, as well as active work between Catherine Crane, Jennifer Harper, Elizabeth Nuthall, and others, indicate robust cooperative relationships within this scholarly community.

**Table 1:** The author of the study on the anxiety and depression of primary school students with the most frequent publication.

Rank	Authors	Counts	Co-cited authors	Counts
1	Putwain, David W	7	COHEN J	167
2	Ginsburg, Golda S	7	HU LT	151
3	Mammarella, Irene C	5	HEMBREE R	140
4	Pekrun, Reinhard	4	ASHCRAFT MH	134
5	Rozelle, Scott	4	RAMIREZ G	127
6	Griffiths, Mark D	4	KESSLER RC	122
7	Ingles, Candido J	4	PEKRUN R	121
8	Ramirez, Gerardo	4	MARSH HW	119
9	Passolunghi, Maria Chiara	4	WIGFIELD A	118
10	Cross,Donna	4	BANDURA A	118

### 3.3 Analysis of country

**Figure 4:**
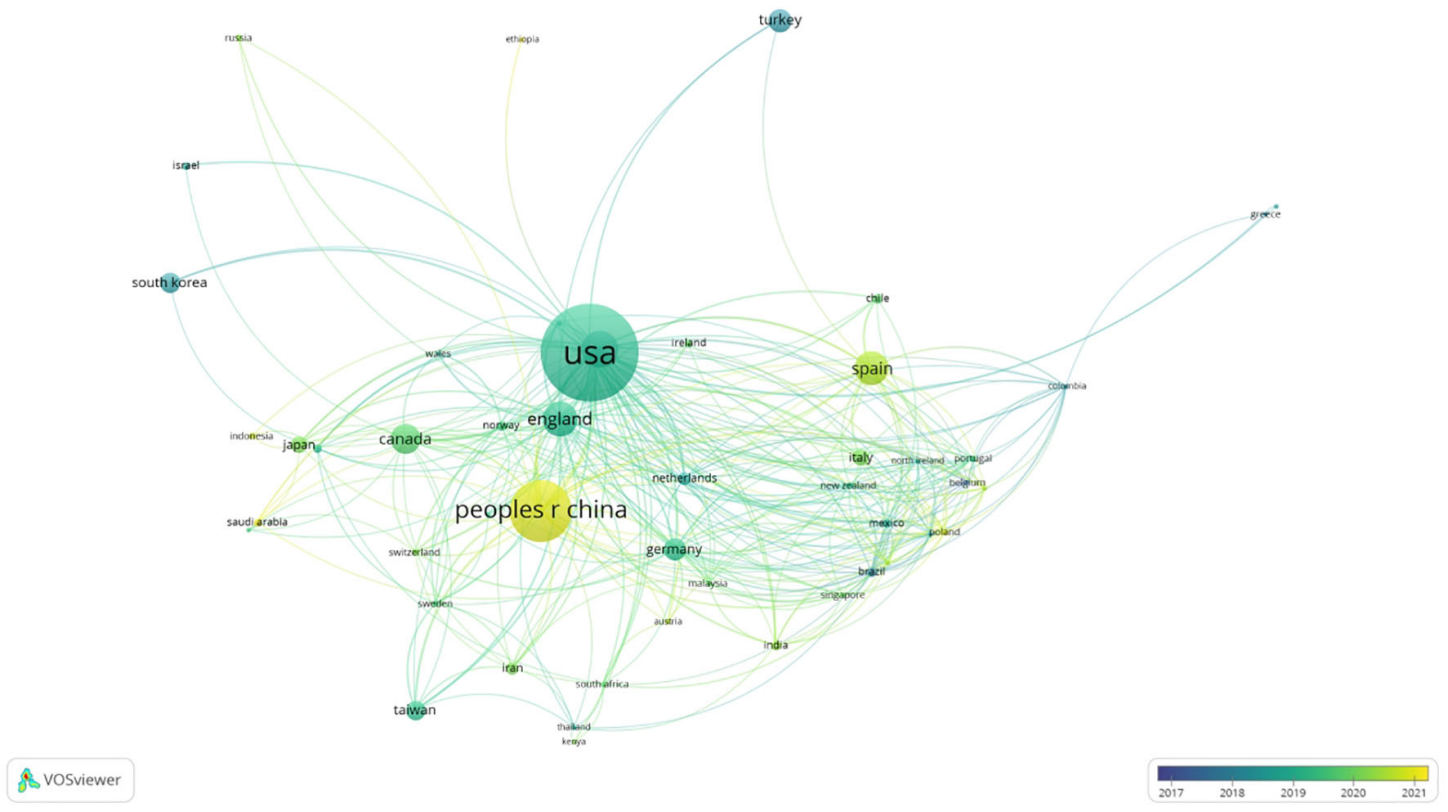
The global distribution of anxiety and depression among primary school students.

As illustrated in **Figure 4**, the leading three countries in terms of publication volume have been identified as the USA (482 publications, 26.91%), China (272 publications, 15.19%), and Australia (141 publications, 7.87%). In terms of centrality, the USA (0.37), England (0.18), and Australia (0.09) are ranked as the top three, respectively. The graphical analysis has revealed a global distribution of research literature on anxiety and depression among primary school students, with dense interconnections between countries, indicating a robust international collaboration network and highlighting extensive cooperative relationships in this research area across numerous countries.

### 3.4 Analysis of institution

**Figure 5:**
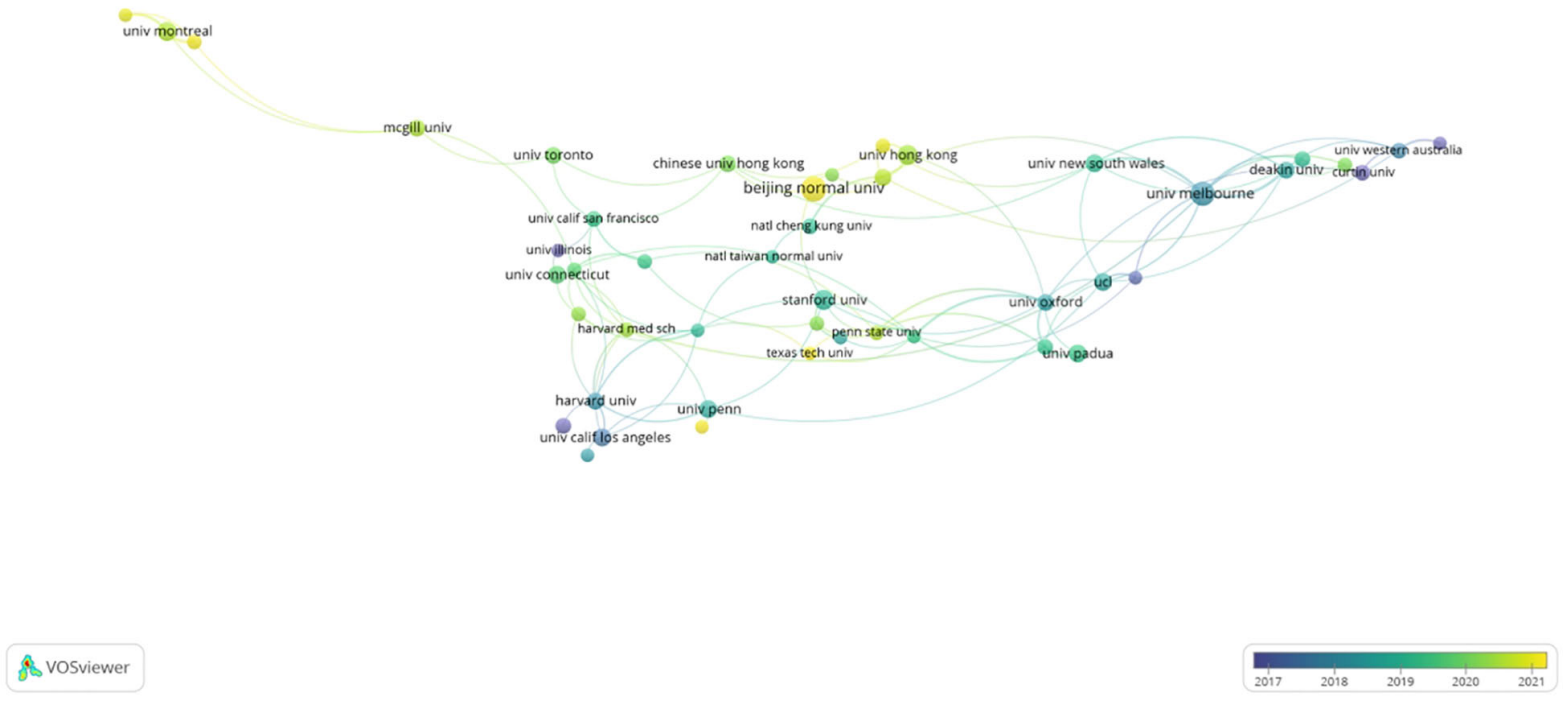
The visualization of organizations of anxiety and depression among primary school students.

The top five cited institutions have been identified as the University of California System, University of London, University of Melbourne, Beijing Normal University, and State University System of Florida (**Table 2**). These institutions are depicted as pivotal nodes within the global cooperation network, engaging in extensive collaborations both among themselves and with other global entities. They are actively involved in research on anxiety and depression among primary school students, yielding significant outcomes. Additionally, in the institution co-occurrence knowledge map (**Figure 5**) illustrates that institutions both domestic and international have established broad cooperative relationships, highlighting the global recognition and attention garnered by this research.

**Table 2:** Top 10 countries and organizations on the research of anxiety and depression among primary school students.

Rank	Country	Counts	Density	Rank	Institution	Counts	Density
1	USA	482	0.37	1	University of California System	61	0.26
2	PEOPLES R CHINA	272	0.06	2	University of London	35	0.1
3	AUSTRALIA	141	0.09	3	Beijing Normal University	30	0.07
4	ENGLAND	132	0.18	4	University of Melbourne	28	0.14
5	SPAIN	126	0.06	5	State University System of Florida	25	0.08
6	CANADA	107	0.1	6	Harvard University	23	0.12
7	TURKEY	78	0	7	Pennsylvania Commonwealth System of Higher Education (PCSHE)	19	0.05
8	GERMANY	76	0.02	8	University College London	18	0.04
9	SOUTH KOREA	65	0	9	Pennsylvania State University	16	0.02
10	TAIWAN	63	0	10	University of Hong Kong	16	0.02

### 3.5 Analysis of references

**Figure 6:**
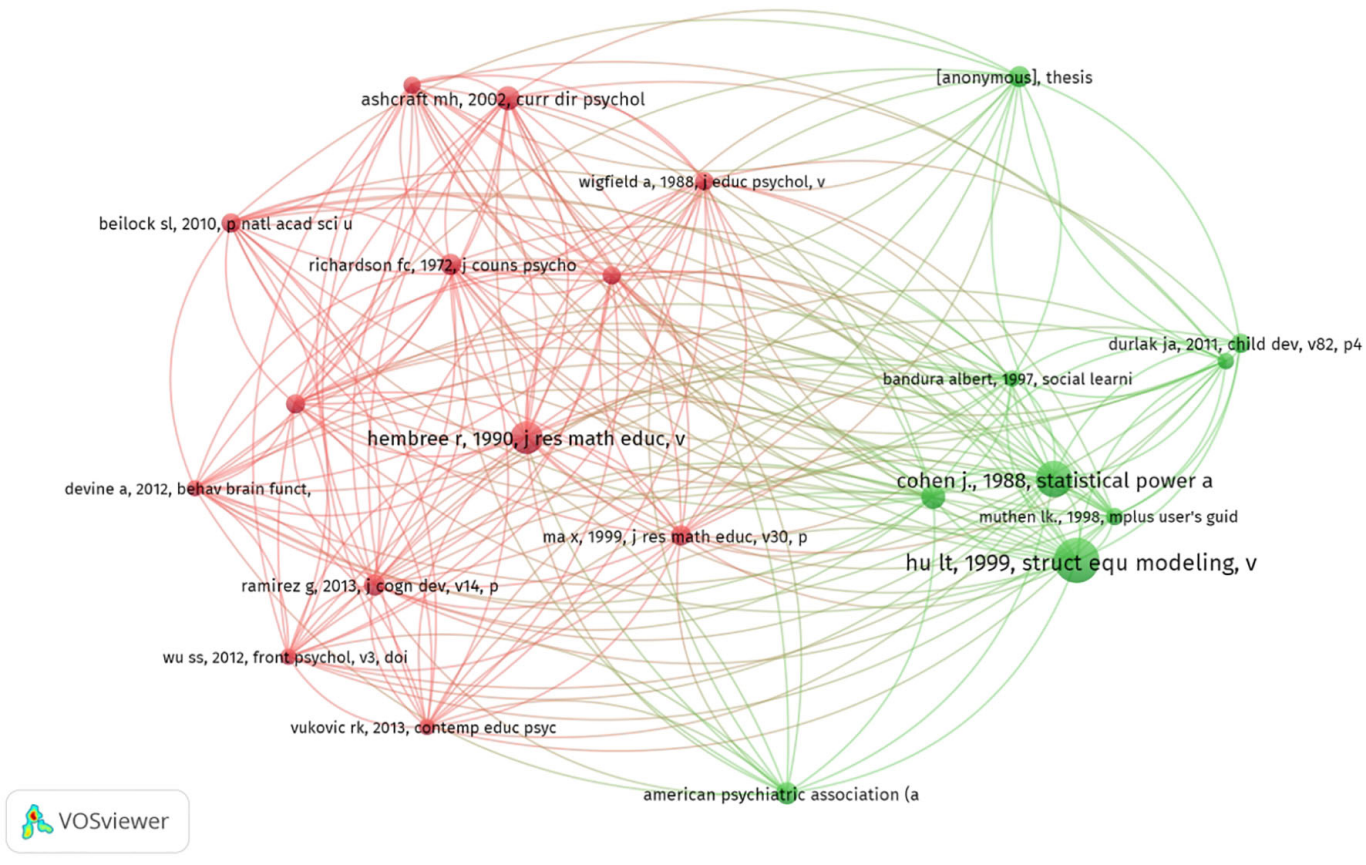
The visualization of co–cited references of anxiety and depression among primary school students.

A co-cited reference is one that appears jointly in multiple publications, thereby establishing it as a foundational element within a specific research domain (13). Among the top ten co-cited references, the most cited article was cited 139 times, the least 61 times, and the average number of citations was 83 times(**Table 3**). As shown in **Figure 6**, “hu lt, 1999, struct equ modelling” demonstrated robust co–citation relationships with “hembree r, 1990, j res math educ”, “richardson fc, 1972, j couns psychol”, “ramirez g, 2016, j exp child psychol”.

**Table 3:** Top 10 co-cited references on the research of anxiety and depression among primary school students.

Rank	Co-cited reference	Counts
1	Hu, L., & Bentler, P. M. (1999). Cutoff criteria for fit indexes in covariance structure analysis: Conventional criteria versus new alternatives. Structural Equation Modeling: A Multidisciplinary Journal, 6(1), 1–55. https://doi.org/10.1080/10705519909540118	139
2	Cohen j., 1988, statistical power analysis for the behavioral sciences	114
3	Hembree, R. (1990). The Nature, Effects, and Relief of Mathematics Anxiety. Journal for Research in Mathematics Education, 21, 33-46. https://doi.org/10.2307/749455.	102
4	Ashcraft, M. H. (2002). Math Anxiety: Personal, Educational, and Cognitive Consequences. Current Directions in Psychological Science, 11(5), 181-185. https://doi.org/10.1111/1467-8721.00196	76
5	Pekrun, Reinhard. (2006). The Control-Value Theory of Achievement Emotions: Assumptions, Corollaries, and Implications for Educational Research and Practice. Educational Psychology Review. 18. 315-341. 10.1007/s10648-006-9029-9.	75
6	american psychiatric association (apa), 2013, diagnostic and statistical manual of mental disorders, doi 10.1176/appi.books.9780890425596	70
7	Richardson, F. C., & Suinn, R. M. (1972). The mathematics anxiety rating scale: psychometric data. Journal of Counseling Psychology, 19(6), 551-554.	66
8	Ramirez, G., Gunderson, E. A., Levine, S. C., & Beilock, S. L. (2013). Math anxiety, working memory, and math achievement in early elementary school. Journal of Cognition and Development, 14(2), 187–202. https://doi.org/10.1080/15248372.2012.664593	65
9	Ma, X. (1999). A Meta-Analysis of the Relationship between Anxiety toward Mathematics and Achievement in Mathematics. Journal for Research in Mathematics Education, 30(5), 520–540. https://doi.org/10.2307/749772	64
10	Ramirez, G., Chang, H., Maloney, E.A., Levine, S.C., & Beilock, S.L. (2016). On the relationship between math anxiety and math achievement in early elementary school: The role of problem solving strategies. Journal of experimental child psychology, 141, 83-100.	61

**Figure 7:**
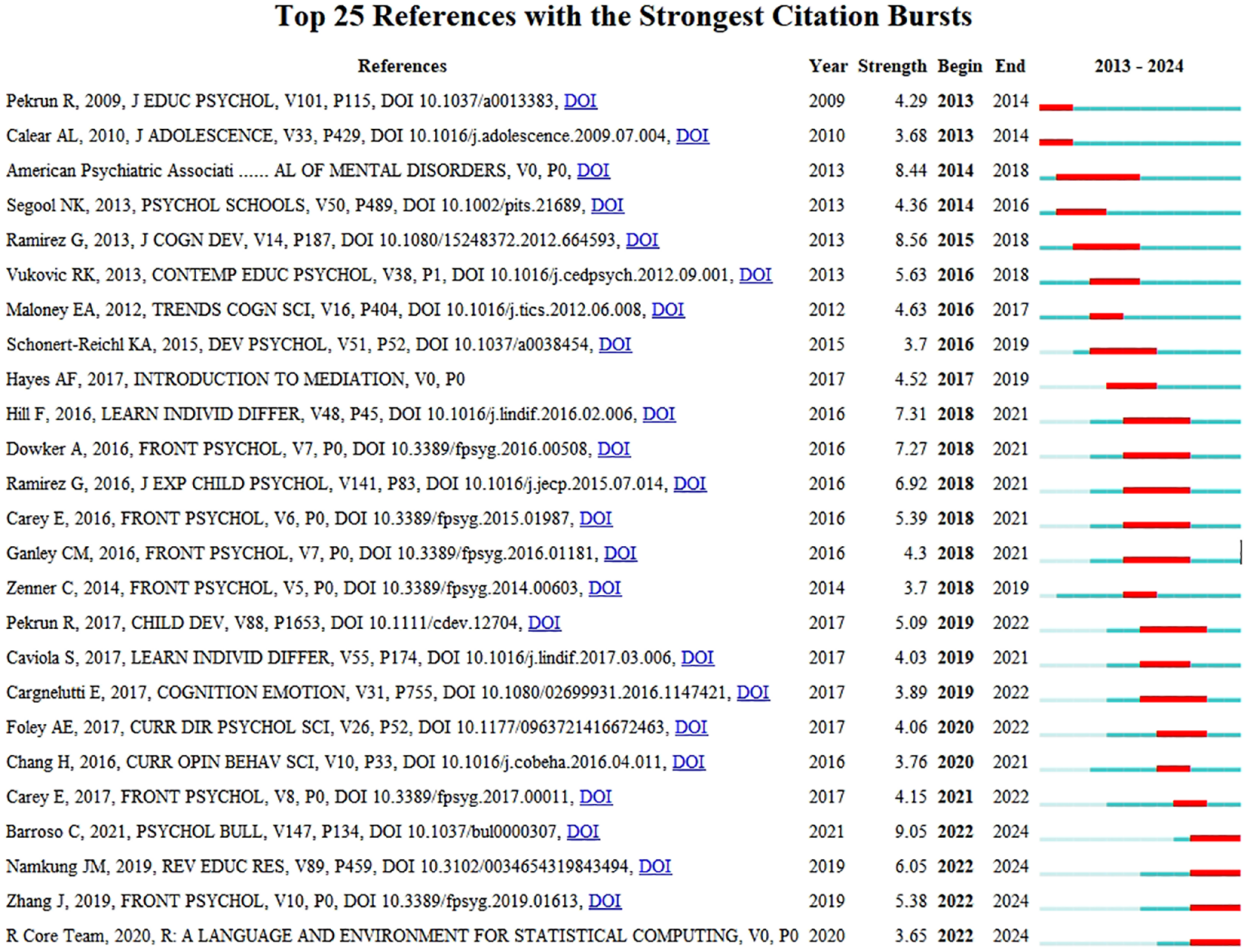
Top 25 references with strong citation bursts of anxiety and depression among primary school students.

The dynamic characteristics of a research topic are manifested by a significant increase in the frequency of citations in the literature. These highly cited documents, referred to as ‘burst literature’, represent the current hotspots in academic research within their respective fields. In CiteSpace software, the display option “Burstness” is configured and initiated by clicking “View” to identify significant citation bursts within the literature on anxiety and depression among primary school students. In the emergence map of cited literature, the red line segment represents the explosive citation time of the corresponding year (**Figure 7**).

### 3.6 Analysis of keywords

Keywords are the essence and focal points of an article, encapsulating its content and key themes. In a specific field, the prominence of a keyword, as indicated by its co-occurrence frequency and centrality, reflects its significance as a research hotspot.

By analyzing keywords, we can swiftly identify the evolving frontiers and hotspots in the research on anxiety and depression among primary school students. In this domain, notably, ‘mental health’ emerges as the most frequently mentioned term, alongside ‘depression’, ‘children’, ‘adolescents’, and ‘anxiety’, which collectively delineate the principal research directions in this field.

**Figure 8:**
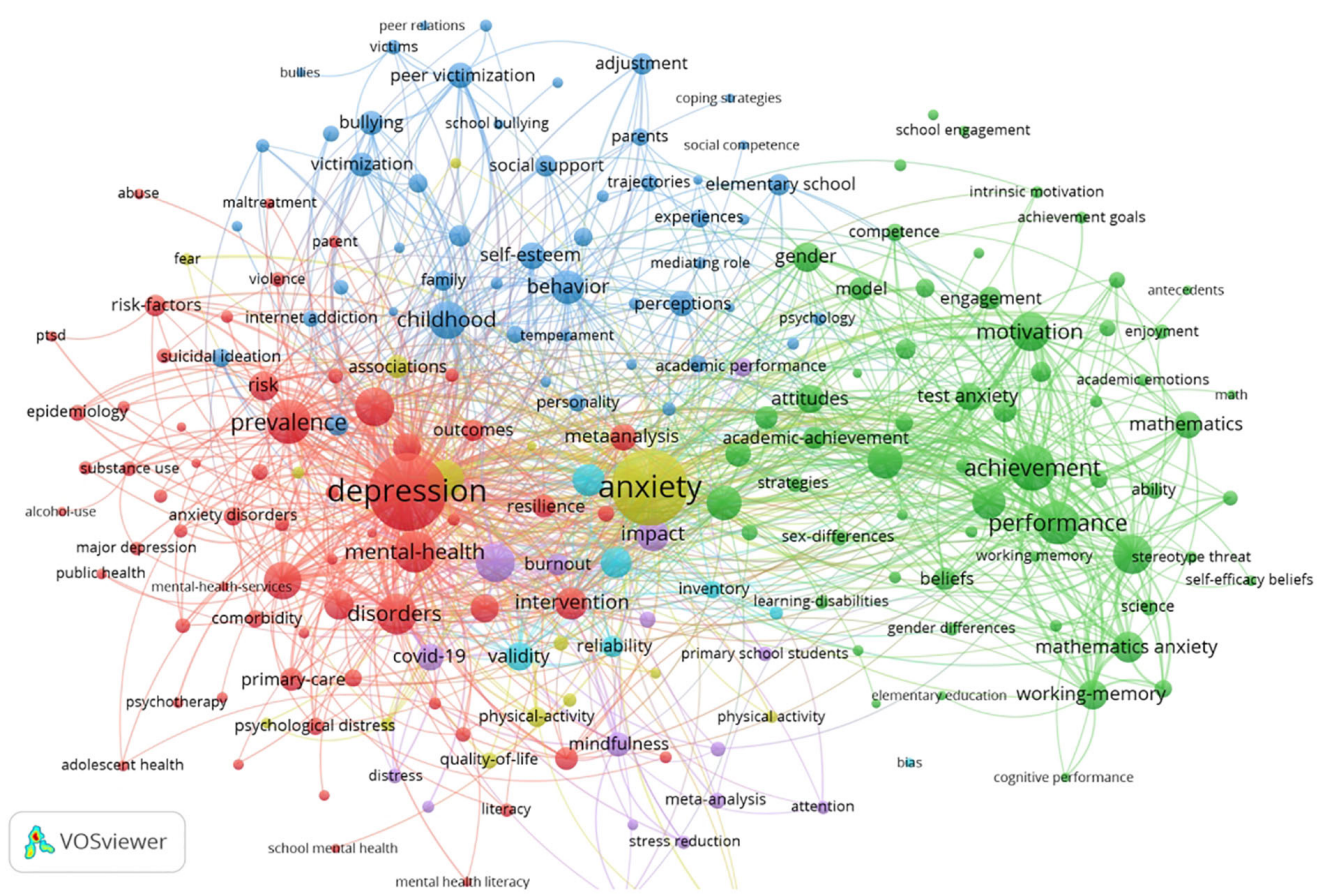
The visualization of frequency keywords on research anxiety and depression among primary school students.

**Figure 9:**
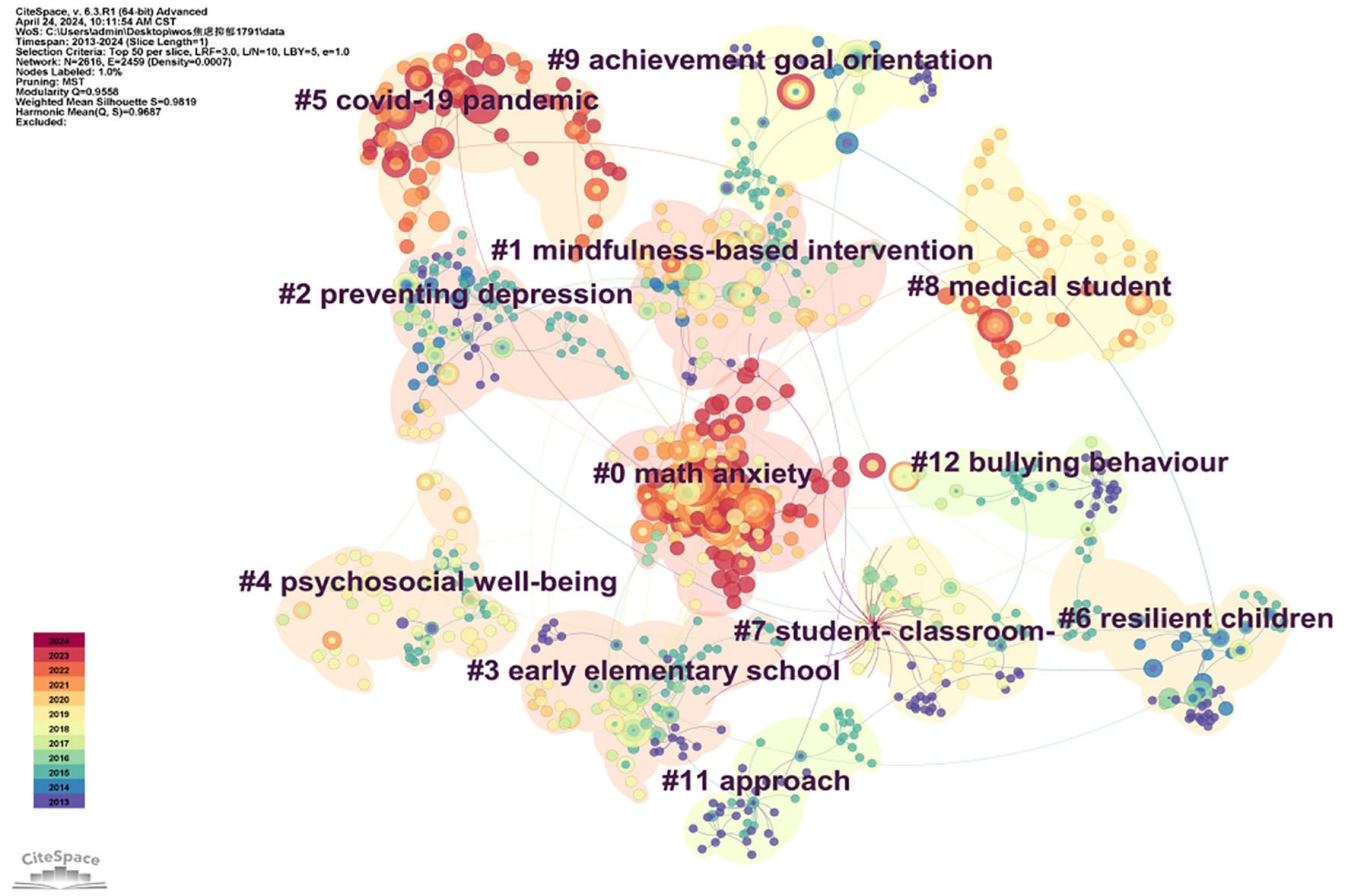
Keywords cluster analysis co-occurrence map.

Using VOSViewer and CiteSpace for visual keyword cluster analysis, the results depicted in **Figure 8** reveal three distinct clusters representing specific research directions. The keywords in the red cluster focus on interventions for anxiety and depression, the blue cluster addresses the mental and behavioral effects of these conditions, and the green cluster explores their formative factors. These clusters underscore the primary research themes: interventions, mental and behavioral impacts, and causative factors of anxiety and depression in primary school students. As a complement, **Figure 9** illustrates the 11 hot keywords in the field of anxiety and depression in primary school children, which were #0 math anxiety, #1mindfulness-based intervention, #2 preventing depression, #3 early elementary school, #4 psychosocial well-being, #5 COVID-19 pandemic, #6 resilient children, #7 student-classroom, #8 medical student, #9 achievement goal orientation, #11 approach, #12 bullying behavior.

## 4 Discussion

### 4.1 General information

In the first period (2013-2017), 443 articles were published, with an average of 86 per year. In the second period (2018-2023), 1478 articles were published, with an average of 246 per year, which is three times that of the first period. Indicated the research on anxiety and depression of primary school students has become increasingly popular, and anxiety and depression of primary school students has attracted increased attention. This phenomenon may be related to a major report (Global Accelerated Action for the Health of Adolescents: Guidance to support country implementation) published by the World Health Organization in 2023 (14). Additionally, the significant increase in publications during (2018-2023) may be attributed to the outbreak of the COVID-19 pandemic (15, 16). The pandemic has had a profound impact on mental health globally, bringing issues such as anxiety and depression to the forefront of academic and public health discussions.

According to the analysis of authors, Putwain, David W from Liverpool John Moores University and Golda S. Ginsburg from the University of Connecticut, both ranked first with seven publications. Committed to the field of educational psychology, Putwain, David W has recently focused on studying the role of achievement emotions in primary school mathematics, the relationship between test anxiety and emotional disorders in primary school, and methods of protection against test anxiety, offering further guidance for school educators to prevent mental health issues (17–19). Unlike Putwain, David W, Ginsburg, Golda S has conducted research on the role of teachers in student anxiety (20). Furthermore, Ginsburg, Golda S has conducted research on preventing the onset of anxiety disorders in the offspring of anxious parents over the past decade (21). Following Putwain, David W and Ginsburg, Golda S, Irene C. Mammarella from the University of Padua was the third most active author with six publications. Recent studies have focused on the differences in visuospatial memory in children with mathematical learning disabilities and on visuospatial processing in students with non-verbal learning disabilities who do not have an intellectual disability (22, 23).

The United States (482 publications) and China (272 publications) lead globally in the volume of research outputs, significantly outpacing other countries. Among the top 10 research institutions, half are based in the United States, indicating robust national research capabilities. There is notable international cooperation, especially between the United States and countries like China, Canada, the United Kingdom, and Australia. In addition, there are robust connections among developing countries. Particularly notable is the collaboration between India, Thailand, and South Africa, which indicates that Asian scholars place significant emphasis on mental health issues. Furthermore, research in this field reveals regional connections, characterized by close cooperation between neighboring countries. This is exemplified by the Asian collaboration network led by China and the European and American collaboration network led by the United States.

Prominent research institutions, including the University of Melbourne, University of Oxford, and Harvard University, have established substantial collaborative networks, engaging with over seven institutions each. Our analysis reveals that these collaborations predominantly involve partners from developed countries, with significantly fewer cooperative engagements with institutions in developing nations. Furthermore, the pattern of collaboration among these institutions tends to be relatively static. To dismantle research barriers and foster a more inclusive global research environment, we advocate for the strengthening of trans-regional cooperation among institutions worldwide. This approach is essential to ensure a broader and more diverse contribution to the critical field of anxiety and depression among primary school students.

### 4.2 Hot spots and trends

Research hotspots are defined as fields that capture significant academic attention during specific periods, particularly due to their relevance to contemporary issues (13). These areas not only mirror the literature that has engaged scholars but also contribute to a cohesive research network. The clustering of keywords effectively summarizes these hotspots, clearly delineating the prevailing topics within the field of anxiety and depression among primary school students. Each cluster identifies a distinct area of focus, enriching our comprehensive understanding of the domain. Through an analysis of high-frequency keywords and their clustering, we have identified dominant themes such as math anxiety, science anxiety, achievement, mindfulness, physical activity, bullying, intervention, COVID-19, suicidal ideation, and gender differences. These themes currently shape the research trends in anxiety and depression among primary school students, highlighting the field’s dynamic and evolving nature.

### 4.3 Intervention

Intervention is a frequent high-frequency word and a trend topic from 2013 to 2020. Among the interventions, mindfulness and physical activities are recognized for their efficacy as gentle treatment options. The emergence of depression and anxiety typically during childhood or adolescence underscores the importance of early intervention.

Research into intervention strategies reveals ongoing exploration with diverse approaches and variable efficacy. A 2013 evaluation indicated that the AOPTP program did not alleviate anxiety and depression in primary school students, though it significantly reduced ADHD prevalence (24). In contrast, a 2015 study demonstrated that school-based cognitive-behavioral therapy (CBT) interventions significantly ameliorated test anxiety, with the most effective strategies being a blend of skills-based and either behavioral or cognitive therapies (25). The educational setting has been identified as an advantageous venue for implementing mental health interventions. A 2017 study assessed the CALM-Child Anxiety Learning Module, a concise nurse-managed intervention based on cognitive-behavioral strategies, noting substantial reductions in anxiety, somatic symptoms, and attentional disturbances (26).In more recent developments, mindfulness-based interventions have gained traction. A 2021 meta-analysis affirmed the effectiveness of these interventions in reducing mild-to-moderate depressive symptoms among adolescents aged 10 to 19 years (27). Further empirical research supports that mindfulness training not only lowers anxiety but also enhances social orientation, positive emotional states, and attentional focus in children (28).

Furthermore, it is noteworthy that the application of artificial intelligence (AI) for the early identification and intervention of anxiety disorders and depression has gained widespread popularity (29). AI applications such as chatbots and virtual assistants conduct initial screenings and symptom assessments through personalized interactions, while wearable and mobile sensors collect objective data like sleep duration, activity levels, and heart rate to inform treatment plans (30, 31). AI algorithms analyze this data to propose personalized treatment strategies, and remote monitoring and support systems aid in detecting depression and providing continuous support, thereby enhancing treatment adherence and engagement (32). Additionally, AI-driven digital therapeutic interventions offer cognitive behavioral therapy (CBT), mindfulness practices, and other evidence-based methods for self-managing depression and anxiety (33, 34). Despite these advancements, challenges remain, including issues of accountability, the need for standardized ethical and legal frameworks, and concerns over data privacy (31). Addressing these challenges is crucial for the responsible and effective use of AI in mental health interventions.

### 4.4 Suicidal ideation

In light of the substantial body of research, it is evident that suicidal ideation (SI) among elementary school students is a critical concern, particularly as it serves as a precursor to suicide attempts and completions. Previous literature identifies SI as a key predictor for such outcomes, emphasizing the need for early and effective intervention (35). The multifaceted nature of risk factors for suicide includes substance abuse, early childhood trauma, stigma associated with seeking help, barriers to accessing care, and availability of means to commit suicide (36). A significant independent factor contributing to SI in children is a contentious home environment children from such backgrounds are at a 3.7 times higher risk of developing SI compared to their peers from harmonious homes. This risk increases dramatically to 27 times in depressed children living in discordant homes compared to non-depressed children in harmonious settings (37). Furthermore, gender-specific analyses reveal that girls typically exhibit higher rates of SI, particularly when exposed to high levels of perceived environmental stress, authoritarian parenting styles, and multiple stressful life events (38).

The correlation between depression and SI is notably strong, with anxiety and sleep disturbances contributing indirectly through their impact on depression (39). Additionally, the relationship between academic and social anxiety and SI underscores the importance of supportive educational and familial environments in mitigating these risks. Conversely, factors such as self-esteem, life satisfaction, and academic achievement serve as protective buffers against the development of SI (40).

Given these insights, it is recommended that schools implement robust support systems to prevent SI in students experiencing high stress or depression. Parents and teachers should vigilantly monitor for any signs of emotional distress and educate children on how to alleviate worries and grievances. Additionally, promoting core self-evaluations in students could serve as a preventive measure against SI, as higher self-esteem and self-worth are associated with lower risks of depression and suicidal thoughts.

### 4.5 COVID-19

COVID-19 is the hot key word for 2020-2023. COVID-19 triggered a pandemic just months after it was first reported in 2019, with more than 774 million confirmed cases globally by 4 February 2024 (41). During the COVID-19 pandemic, the prevalence of anxiety and depression among students has increased due to the lockdown policy and panic without specific drugs. According to the research, during the COVID-19 pandemic, 88.4% of students have experienced anxiety, 72.1% have been diagnosed with depression, and 35.7% have experienced moderate to severe stress (42). These issues were particularly acute among females, older students, and those from larger or low-income families, exacerbated by pandemic-related economic and educational disruptions (3). The correlation between increased anxiety and depression symptoms and pandemic stressors is clear, while social support has proven to mitigate these effects (43). The transition to online learning and reduced social interactions further compounded these challenges, affecting students’ mental well-being and their ability to adapt to traditional learning environments (44). Addressing these issues requires a collaborative approach involving educational institutions, healthcare providers, and policymakers to integrate comprehensive mental health strategies within educational settings. This includes enhancing online learning environments with mental health resources, improving access to psychological counseling, and building robust community support systems to help young learners navigate these unprecedented challenges effectively.

### 4.6 Bullying

bullying is a serious global issue within the educational sector, affecting not only the academic achievements and social capabilities of victims but also inflicting profound psychological impacts on children (45). Bullying behavior has been extensively studied and linked to a variety of psychological health issues. Of particular concern is the relationship between bullying and anxiety and depression among primary school students, which has garnered widespread attention in the field of mental health. In 2018, Bayer conducted a survey across numerous primary schools in Australia, revealing that a significant 29% of students frequently faced bullying, with physical bullying affecting 13.8% and verbal bullying 22.7% of students (46). Subsequent research by Shayo linked bullying to increased instances of suicide, identifying it as a significant predictor, especially among victims exhibiting suicidal ideation and a heightened likelihood of attempting suicide (47). Complementarily, Diana’s study established a positive correlation between exposure to school bullying and the development of depression, significantly highlighting that bullying escalates the risk of depression in primary school students (48). Further investigations have shown that bullying victims also suffer from higher levels of anxiety, Internet gaming disorder, and mobile phone addiction (49). These findings underscore the critical need for comprehensive anti-bullying strategies that involve families, schools, and societal interventions to effectively mitigate the adverse mental health impacts of bullying on children.

### 4.7 Future research trend

#### 4.7.1 Math anxiety

Math anxiety, a complex state elicited by math-related stimuli, represents a considerable source of distress among elementary students, encompassing cognitive, emotional, behavioral, and physiological aspects (50). Notably, this anxiety is widespread among primary students, whose neural development is not yet fully mature, making them especially vulnerable to anxiety when grappling with abstract concepts. A study involving 1,327 children from grades 2 to 5 revealing that over 15% reported experiencing math anxiety (51). The research further indicates that math anxiety emerges from the early stages of schooling, with first-grade students exhibiting mild anxiety that escalates at the beginning of the academic year; while the majority of children experience low levels of math anxiety, a minority report higher levels, often linked to the fear of failure, task difficulty, time pressure, and concerns over poor grades (52).However, Nathan conducted a global survey across various countries and age groups, uncovering significant disparities in the relationship between math anxiety and mathematics performance, thereby highlighting the crucial role of educational and cultural backgrounds in comprehending the impact of math anxiety on academic achievement (53). Contextual and linguistic teaching methods have been found to evoke math anxiety less than traditional symbol-based instruction, suggesting that the teaching approach plays a role in the development of this anxiety (54). An eye-tracking study investigating the inner workings of math anxiety revealed that students with this condition frequently exhibit inadequate attention control when solving math problems (55). Gender differences in math anxiety have also been documented, with Perez’s research demonstrating that girls generally exhibit higher levels of math anxiety than boys, a gap that increases with age (56). These findings underscore the multifaceted nature of math anxiety and the necessity for tailored educational strategies to address it effectively.

### 4.8 Advantages and shortcomings

The bibliometric analysis conducted in this study demonstrates unique strengths. Firstly, there is currently a lack of research employing bibliometric methods to address anxiety and depression issues among elementary school students. This study examines the research landscape surrounding multimodal imaging tools using bibliometric methods. We utilized two different types of bibliometric software for bibliometric and visual analysis, synthesizing relevant publications on anxiety and depression among elementary school students over the past decade. The entire analytical process was conducted rigorously and objectively, resulting in credible findings. Additionally, this systematic analysis provides comprehensive guidance to scholars in this research domain, offering a more objective and comprehensive presentation of research hotspots and trends compared to traditional reviews, along with predictions for future research directions. It is important to note that this study solely relied on the Web of Science Core Collection as its source of literature, which may introduce certain limitations in terms of literature sources. Additionally, the use of bibliographic co-citation analysis inherently poses some challenges, particularly regarding the citation frequency of newly published papers. To address this shortcoming, this paper analyzes and summarizes the hot spots and emerging trends of anxiety and depression among primary school students by using keyword co-occurrence clustering. Future studies, while ensuring the quality of literature data, could expand the scope of data retrieval, innovate in related analytical methods, and strive for a more complete and accurate portrayal of research progress in this field.

## 5 Conclusion

The escalating number of articles published annually on elementary students’ anxiety underscores the intensifying global focus on this issue. Putwain, David W., is the most prolific author in this field, while COHEN J. is the most cited. The United States and China dominate the publication landscape in this field, yet their collaborations are predominantly with economically and technologically advanced countries. To elevate the caliber of global collaborative research, fostering enhanced cooperation among nations and institutions is crucial.

Present investigations into anxiety and depression among elementary students concentrate on intervention strategies, mental health, and the precipitating factors and intrinsic mechanisms of these conditions. Current research highlights include studies on suicidal ideation, bullying, the effects of COVID-19, and mindfulness interventions. Future research is poised to delve into the impact of mathematical anxiety on the psychological health and behavioral patterns of primary school students. Additionally, our findings indicate that family stress and academic burden are significant contributors to the prevalence of anxiety and depression among primary school students. There is notable heterogeneity across different groups and cultures. Specifically, in Asian populations, academic burden are identified as the primary factors leading to anxiety and depression in primary school students. This study not only furnishes a benchmark for current hot topics and emerging frontiers in the realm of elementary students’ anxiety and depression but also forecasts pivotal research trends, thereby providing valuable guidance for ongoing scholarly inquiry.

## Author contributions

JF: Writing – original draft, Software, Investigation, Conceptualization. WY: Writing – review & editing, Supervision, Methodology, Data curation. SL: Writing – review & editing, Supervision, Data curation. WS: Writing – review & editing, Supervision, Methodology, Data curation.
